# Quantification of myocardial scar of different etiology using dark- and bright-blood late gadolinium enhancement cardiovascular magnetic resonance

**DOI:** 10.1038/s41598-024-52058-8

**Published:** 2024-03-05

**Authors:** Lamis Jada, Robert J. Holtackers, Bibi Martens, Hedwig M. J. M. Nies, Caroline M. Van De Heyning, Rene M. Botnar, Joachim E. Wildberger, Tevfik F. Ismail, Reza Razavi, Amedeo Chiribiri

**Affiliations:** 1 School of Biomedical Engineering and Imaging Sciences, King’s College London, St Thomas’ Hospital, London, United Kingdom; 2 King Abdulaziz University, Jeddah, Kingdom of Saudi Arabia; 3 Cardiovascular Research Institute Maastricht (CARIM), Maastricht University, Maastricht, The Netherlands; 4 Department of Radiology and Nuclear Medicine, Maastricht University Medical Centre, Maastricht, The Netherlands; 5 GENCOR, University of Antwerp, Antwerp, Belgium; 6 Department of Cardiology, Antwerp University Hospital, Antwerp, Belgium; 7 Institute for Biological and Medical Engineering, Pontificia Universidad Católica de Chile, Santiago, Chile; 8 Millennium Institute for Intelligent Healthcare Engineering, Santiago, Chile

**Keywords:** Cardiology, Biomedical engineering

## Abstract

Dark-blood late gadolinium enhancement (LGE) has been shown to improve the visualization and quantification of areas of ischemic scar compared to standard bright-blood LGE. Recently, the performance of various semi-automated quantification methods has been evaluated for the assessment of infarct size using both dark-blood LGE and conventional bright-blood LGE with histopathology as a reference standard. However, the impact of this sequence on different quantification strategies in vivo remains uncertain. In this study, various semi-automated scar quantification methods were evaluated for a range of different ischemic and non-ischemic pathologies encountered in clinical practice. A total of 62 patients referred for clinical cardiovascular magnetic resonance (CMR) were retrospectively included. All patients had a confirmed diagnosis of either ischemic heart disease (IHD; n = 21), dilated/non-ischemic cardiomyopathy (NICM; n = 21), or hypertrophic cardiomyopathy (HCM; n = 20) and underwent CMR on a 1.5 T scanner including both bright- and dark-blood LGE using a standard PSIR sequence. Both methods used identical sequence settings as per clinical protocol, apart from the inversion time parameter, which was set differently. All short-axis LGE images with scar were manually segmented for epicardial and endocardial borders. The extent of LGE was then measured visually by manual signal thresholding, and semi-automatically by signal thresholding using the standard deviation (SD) and the full width at half maximum (FWHM) methods. For all quantification methods in the IHD group, except the 6 SD method, dark-blood LGE detected significantly more enhancement compared to bright-blood LGE (*p* < 0.05 for all methods). For both bright-blood and dark-blood LGE, the 6 SD method correlated best with manual thresholding (16.9% vs. 17.1% and 20.1% vs. 20.4%, respectively). For the NICM group, no significant differences between LGE methods were found. For bright-blood LGE, the 5 SD method agreed best with manual thresholding (9.3% vs. 11.0%), while for dark-blood LGE the 4 SD method agreed best (12.6% vs. 11.5%). Similarly, for the HCM group no significant differences between LGE methods were found. For bright-blood LGE, the 6 SD method agreed best with manual thresholding (10.9% vs. 12.2%), while for dark-blood LGE the 5 SD method agreed best (13.2% vs. 11.5%). Semi-automated LGE quantification using dark-blood LGE images is feasible in both patients with ischemic and non-ischemic scar patterns. Given the advantage in detecting scar in patients with ischemic heart disease and no disadvantage in patients with non-ischemic scar, dark-blood LGE can be readily and widely adopted into clinical practice without compromising on quantification.

## Introduction

Late gadolinium enhancement (LGE) cardiovascular magnetic resonance (CMR) is considered the method of choice for the identification of areas of myocardial scar in patients with known or suspected cardiac disease^1,2^. Standard LGE acquisitions are typically based on inversion-recovery (IR) pulse sequences where the inversion time (TI) is set to null signal from the normal myocardium. This widely-adopted method results in the acquisition of bright-blood LGE images^3,4^. It has been shown, however, that bright-blood LGE may underestimate the extent of ischemic subendocardial scar due to the proximity between the scar and the often similarly bright left ventricular (LV) blood pool. To overcome this limitation, various novel techniques have been proposed that enable the acquisition of “dark-blood” LGE images^5^. Most of these techniques, however, require extra radiofrequency pulses for additional magnetization preparation to suppress or reduce the signal intensity of the blood pool, and therefore require the installation and optimization of new acquisition sequences or scanner software^6–8^.

An exception, however, is a previously proposed dark-blood LGE method^9^ which does not require additional magnetization preparation. This method is based on a standard phase-sensitive inversion-recovery (PSIR) sequence that is acquired using a short TI to null the blood pool signal instead of the signal of normal myocardium. Blood-nulled PSIR LGE achieves higher scar-to-blood contrast compared to conventional bright-blood LGE while still maintaining good contrast between scar and normal myocardium^9,10^. This approach allows for an improved detection and visualization of areas of ischemic subendocardial scar compared to standard bright-blood LGE and can be acquired on CMR scanners from different vendors, at different field-strengths, using a standard PSIR sequence^11^. More recently, blood-nulled PSIR LGE imaging has been validated against histology in a swine model of myocardial infarction (MI) and showed better agreement with histopathology in comparison to standard bright-blood LGE imaging^12^.

Whilst visual assessment is routinely utilized for clinical reporting, LGE quantification is commonly used to measure the extent of LGE for research purposes^13^ and may play a role in risk stratification for some cardiomyopathies^14^. In the past 20 years, various methods for LGE quantification have been proposed and compared, ranging from simple manual contouring to more advanced (semi-)automated methods, including signal intensity-based thresholding using the standard deviation (SD) and the full width at half maximum (FWHM) methods.

So far, however, quantification of LGE has mainly been applied to conventional bright-blood LGE images, or to dark-blood LGE images but then excluding non-ischemic patterns of scar.

Given the higher sensitivity and greater conspicuity of ischemic scar on dark-blood sequences, it cannot be assumed that quantification methods employed for research and clinical practice will perform in an equivalent way. We therefore compared various LGE quantification methods using both dark- and bright-blood LGE in different groups of patients with both ischemic and non-ischemic pathologies commonly encountered in clinical practice.

## Methods

### Study population

A total of 62 patients with scar on LGE imaging and referred for clinical CMR between February and September 2021 were retrospectively included. These patients included subjects with ischemic heart disease (IHD; n = 21), dilated/non-ischemic cardiomyopathy (NICM; n = 21) including patients with dilated cardiomyopathy (DCM) or previous myocarditis, and hypertrophic cardiomyopathy (HCM; n = 20).

### CMR protocol

All patients underwent CMR on a 1.5 T scanner (Somatom Aera; Siemens Healthineers, Erlangen, Germany) and imaging was performed according to the standardized protocols recommended by the Society for Cardiovascular Magnetic Resonance (SCMR)^15^. Scout images and long-axis cine images were obtained first, followed by an intravenous injection of 0.15 mmol/kg gadobutrol (Gadovist; Bayer Pharmaceuticals, Berlin, Germany) and in any case not exceeding a maximum volume of 20 mL even in patients with raised body weight, in accordance with institutional guidelines. A short-axis cine stack for volumetric measurements was then acquired immediately after gadolinium injection. Starting from ten minutes post-injection, both bright-blood and dark-blood LGE imaging were acquired in randomized order using a standard PSIR LGE sequence in full short-axis stack and in three long axis views. The first set of LGE images was acquired starting from 10 min post-injection and the second set of LGE images starting from 20 min post-injection. Typical imaging parameters for the ECG-triggered PSIR LGE sequence with a balanced steady-state free-precession (bSSFP) readout were: echo time (TE) 1.2 ms, repetition time (TR) 2.9 ms, flip angle 45 degrees, reference flip angle 8 degrees, acquired resolution 1.4 × 1.9 mm^2^, and slice thickness 8 mm. Both LGE methods used identical sequence settings apart from the TI parameter, which was set to null the myocardium in bright-blood LGE and to null the LV blood pool in dark-blood LGE respectively. Look-Locker/TI scout scans were performed before each set of LGE acquisitions to determine the optimum TI. All LGE images were acquired in the mid-diastolic resting period during end-expiration breath-holding. The mechanism for the dark-blood LGE method without additional magnetization preparation has been described in earlier work^9^.

### Image analysis

All analyses were performed using clinically approved, commercially available software (CVI42, v.5.12.1, Circle Cardiovascular Imaging, Calgary, Canada). LV and right-ventricular (RV) volumes, and LV mass were measured on cine images according to standardized analysis methods^13^. Short-axis bright- and dark-blood LGE images were manually segmented for the epicardial and endocardial borders, while excluding the papillary muscles. The extent of LGE was then measured visually by manual signal thresholding, and by the semi-automated SD and FWHM threshold methods.

**Figure 1:**
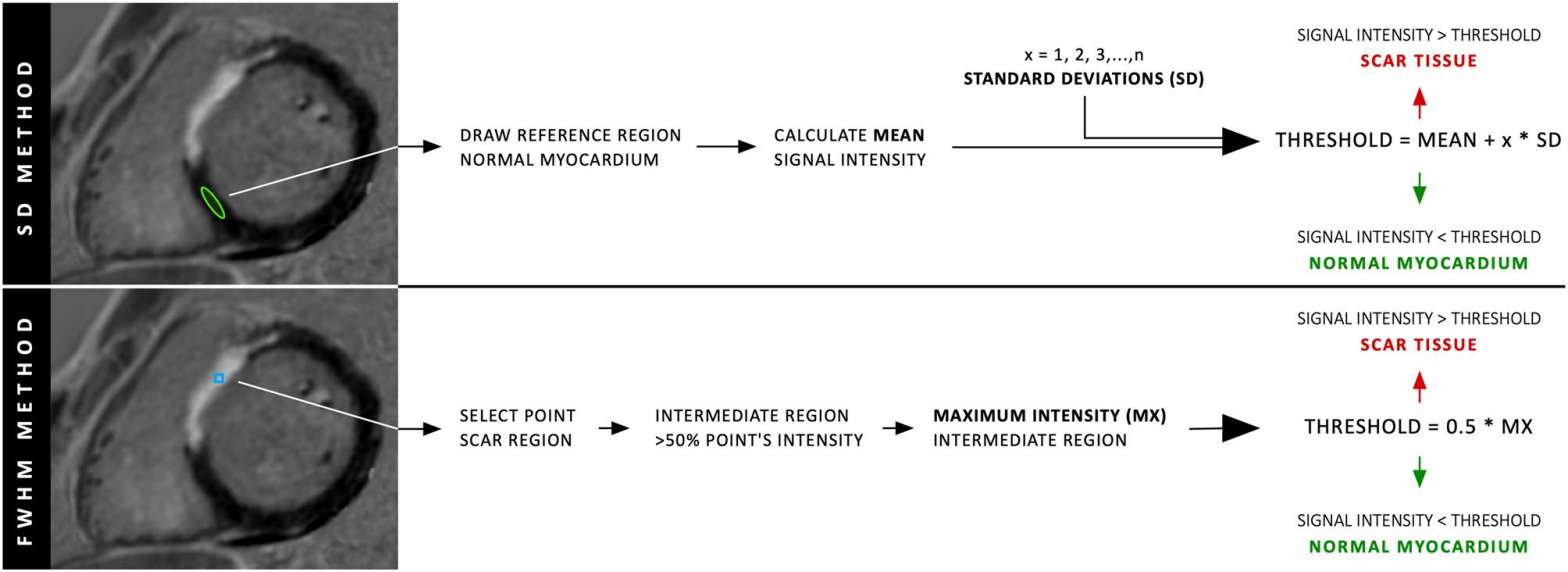
Schematic overview of two semi-automated signal intensity thresholding methods, with the standard deviation (SD) method in the top panel and the full width at half maximum (FWHM) method in the bottom panel.

For the SD method, a region of interest was manually drawn in an area of myocardium visually judged negative for the presence of enhancement to determine the average signal level and SD in remote myocardium (Fig. 1, top panel). The volume of enhancement was then derived for signal thresholds of various number of SDs above the average of the remote myocardial signal. In an earlier study, using thresholds of 3–8 SDs and histopathology as reference standard, it was found that a larger number of thresholds (> 6) underestimates the burden of LGE, and 5 SDs was found most accurate for both dark-blood and bright-blood LGE)^16^. However, there is a rationale for trying a lower number of SDs (< 3) as threshold in non-ischemic disease as the scarred tissue might appear less bright than in ischemic disease. Therefore, signal thresholds of 2 to 6 SDs were used in this study, similar to Flett et al.^17^ who also investigated both ischemic and non-ischemic scar but using bright-blood LGE only. For the FWHM method, a separate point of interest was selected in an area of overt enhancement (Fig. 1, bottom panel). An intermediate region > 50% of that point’s signal intensity was then established. A threshold of 50% between the maximum signal intensity of that intermediate region and the minimum value in the myocardium was used to quantify the volume of enhancement. Bright-blood and dark-blood images were independently and randomly assessed on different days for each quantification method. Results were recorded as the percentage of enhanced LV myocardium by dividing the total enhanced myocardial mass by the total LV mass.

In a subset of 15 patients (stratified to include five patients for each study group), LGE quantification was repeated twice on two different occasions by the same reader and by a different reader to assess both intra-observer and inter-observer variability, respectively.

### Statistical analysis

Within each group of patients and for all used quantification methods (manual, 2 to 6 SDs, and FWHM), both LGE methods were compared using either a paired-sample *t*-test (normally distributed data) or a non-parametric Wilcoxon signed-rank test (non-normally distributed data). Normality of data was evaluated using the Shapiro–Wilk test. Intra- and interobserver variability was assessed by the intraclass correlation coefficient (ICC) using a two-way mixed model set for absolute agreement. All statistical analyses were performed using SPSS (version 27, IBM, Armonk, NY, USA). Two-tailed values of *p* < 0.05 were considered significant.

### Ethical approval and consent to participate

All patients provided written informed consent at the time of the MRI scan for potential inclusion in the study under ethics committee approval 15/NS/0030. The study was conducted according to the principles of the Declaration of Helsinki.

## Results

**Figure 2:**
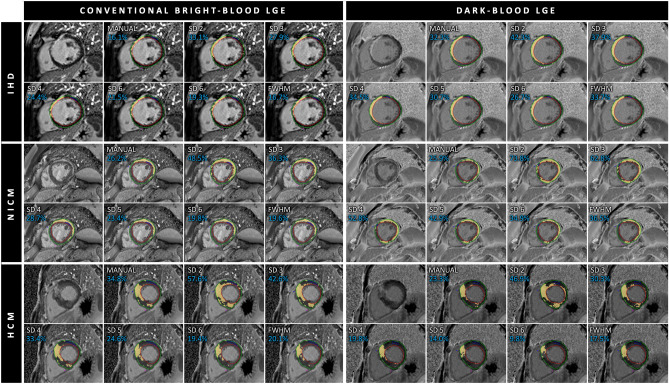
Manual and semi-automated scar quantification in a case of ischemic heart disease (IHD, top rows), dilated/non-ischemic heart disease (NICM, middle rows), and hypertrophic cardiomyopathy (HCM, lower rows) using both conventional bright-blood LGE (left panel) and dark-blood LGE (right panel). The cyan number in each image indicates the % of enhanced left ventricular myocardium for the given method. SD, standard deviation; FWHM, full width at half maximum.

Complete bright-blood LGE and dark-blood LGE datasets were obtained in 62 patients. Table 1 summarizes the demographics and CMR characteristics of the three subgroups. Figure 2 shows imaging examples of the used quantification methods in each patient group using both bright-blood and dark-blood LGE images.

**Table 1:** Demographics and CMR characteristics of the study population.

	IHD (n = 21)	NICM (n = 21)	HCM (n = 20)
Age (years)	61 ± 10	48 ± 16	53 ± 13
Sex (n, m/f)	17/4	17/4	17/3
BSA (m^2^)	2.1 ± 0.3	2.1 ± 0.3	2.0 ± 0.2
LVEDV (mL)	228 ± 67	200 ± 61	155 ± 36
iLVEDV (ml/m^2^)	109 ± 28	98 ± 28	77 ± 20
LVESV (mL)	136 ± 52	101 ± 59	55 ± 18
iLVESV (ml/m^2^)	65 ± 23	49 ± 28	27 ± 9
LVEF (%)	42 ± 8	52 ± 13	65 ± 6
LV mass (g)	127 ± 38	112 ± 35	157 ± 49
iLV mass (g/m^2^)	61 ± 15	54 ± 14	77 ± 22

Data are expressed as mean ± standard deviation unless indicated otherwise. IHD, ischemic heart disease; NICM, dilated/non-ischemic cardiomyopathy; HCM, hypertrophic cardiomyopathy; BSA, body surface area; LVEDV, left ventricular end-diastolic volume; iLVEDV, indexed left ventricular end-diastolic volume; LVESV, left ventricular end-systolic volume; iLVESV, indexed left ventricular end-systolic volume; LVEF, left ventricular ejection fraction.

In the IHD group, the 6 SD method correlated best with manual quantification for both bright-blood and dark-blood LGE (16.9% vs. 17.1% and 20.1 vs. 20.4%, respectively). For all quantification methods in the IHD group (except the 6 SD method), however, dark-blood LGE detected significantly more LV enhancement compared to bright-blood LGE (*p* < 0.05 for all methods). In the NICM group, the 5 SD method correlated best with manual quantification for bright-blood LGE (9.3% vs. 11.0%), while for dark-blood LGE the 4 SD method correlated best (12.6% vs. 11.5%). No significant differences between LGE methods were found for any of the quantification methods in the NICM group. Similarly, for the HCM group, no significant differences between both LGE methods were found for any of the quantification methods. In this group, the 6 SD method correlated best with manual quantification for bright-blood LGE (10.9% vs. 12.2%), while for dark-blood LGE the 5 SD method correlated best (13.2% vs. 11.5%). Although non-significant in the HCM group, dark-blood LGE showed a trend towards quantification of smaller scar sizes compared to bright-blood LGE for all quantification methods.

**Figure 3:**
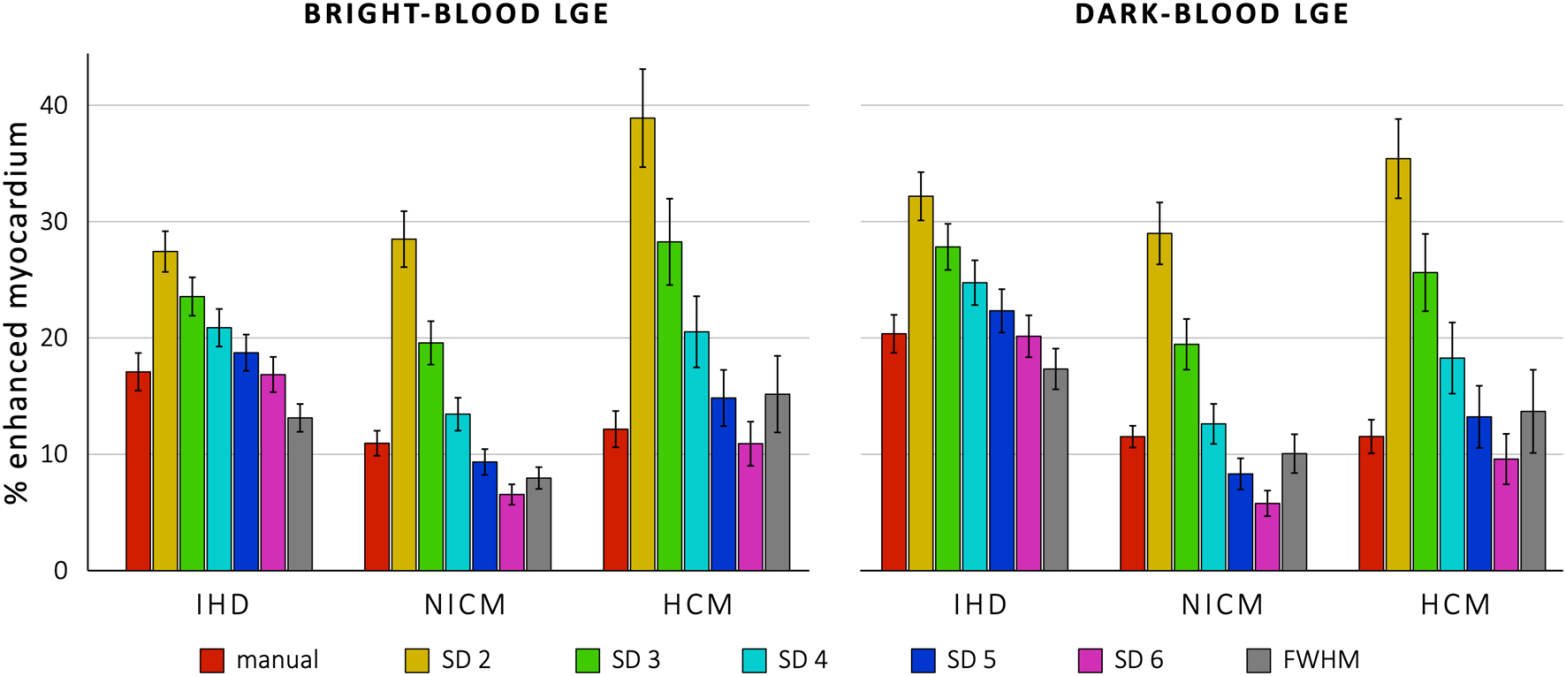
Graphical presentation of the mean percentages of enhanced left ventricular myocardium for the ischemic heart disease (IHD), dilated/non-ischemic cardiomyopathy (NICM), and hypertrophic cardiomyopathy (HCM) groups as assessed by the various quantification methods for both conventional bright-blood LGE (left panel) and dark-blood LGE (right panel). The error bars indicate the standard error of the mean.

**Figure 4:**
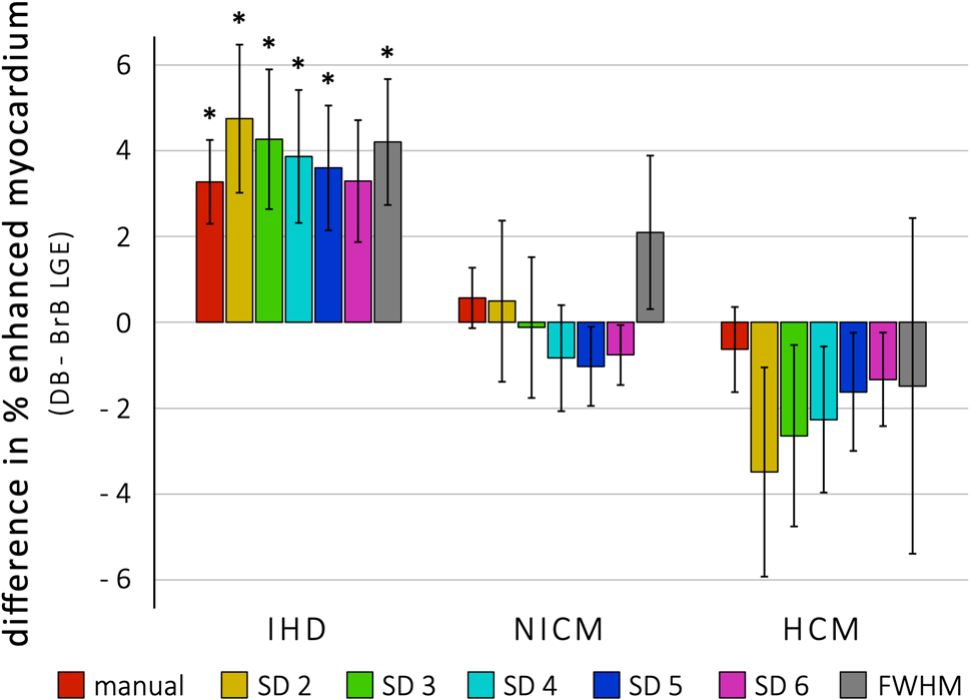
Graphical presentation of the mean differences in percentage of enhanced left ventricular myocardium between dark-blood LGE and conventional bright-blood LGE as assessed by the various quantification methods in the ischemic heart disease (IHD), dilated/non-ischemic heart disease (NICM), and hypertrophic cardiomyopathy (HCM) groups. The error bars indicate the standard error of the mean. The asterisks indicate a statistically significant difference. SD, standard deviation, FWHM, full width at half maximum.

Figure 3 and Table 2 illustrate the mean percentages of LV enhancement as assessed by the various quantification methods for both bright-blood LGE and dark-blood LGE in all three patient groups. Figure 4 illustrates the differences between bright- and dark-blood LGE methods for all quantification methods in the three patient groups.

**Table 2:** The percentage of enhanced left ventricular myocardium for the three patient groups.

	Bright-blood LGE			Dark-blood LGE		
	IHD	NICM	HCM	IHD	NICM	HCM
Manual	17.1 ± 7.4	11.0 ± 4.9	12.2 ± 7.0	20.4 ± 7.5	11.5 ± 4.3	11.5 ± 6.5
SD 2	27.4 ± 8.0	28.5 ± 11.0	38.9 ± 18.8	32.2 ± 9.5	29.0 ± 12.2	35.4 ± 15.2
SD 3	23.6 ± 7.5	19.6 ± 8.5	28.3 ± 16.6	27.8 ± 9.1	19.5 ± 10.0	25.6 ± 14.8
SD 4	20.9 ± 7.4	13.4 ± 6.5	20.5 ± 13.7	24.7 ± 8.8	12.6 ± 7.9	18.3 ± 13.7
SD 5	18.7 ± 7.2	9.3 ± 5.1	14.8 ± 10.8	22.3 ± 8.5	8.3 ± 6.1	13.2 ± 11.9
SD 6	16.9 ± 6.9	6.5 ± 4.0	10.9 ± 8.5	20.1 ± 8.2	5.8 ± 5.0	9.6 ± 9.7
FWHM	13.1 ± 5.5	8.0 ± 4.3	15.2 ± 14.7	17.3 ± 8.0	10.1 ± 7.6	13.7 ± 16.0

Data are expressed as mean ± standard deviation unless indicated otherwise. IHD, ischemic heart disease; NICM, dilated/non-ischemic cardiomyopathy; HCM, hypertrophic cardiomyopathy; SD, standard deviation, FWHM, full width at half maximum.

### Inter- and intra-observer agreement

Regardless of the LGE technique used, inter- and intra-observer agreement was higher in the IHD group compared to the NICM and HCM groups. For the IHD and NICM groups, dark-blood LGE showed on average a higher inter-observer agreement compared to bright-blood LGE. For the HCM group, bright-blood LGE showed on average higher inter-observer agreement. With regard to intra-observer agreement, dark-blood LGE showed on average superior agreement compared to bright-blood LGE in all patient groups. Detailed results on inter- and intra-observer agreement are presented in Table 3.

**Table 3:** The intraclass correlation coefficients (ICCs) as a measure for inter- and intra-observer variability.

	Inter-observer						Intra-observer					
	Bright-blood LGE			Dark-blood LGE			Bright-blood LGE			Dark-blood LGE		
	IHD	NICM	HCM	IHD	NICM	HCM	IHD	NICM	HCM	IHD	NICM	HCM
Manual	0.73	− 0.75	0.40	0.92	0.04	0.18	0.99	0.12	0.83	0.95	0.61	0.85
SD 2	0.73	− 0.19	0.42	0.95	0.42	0.30	0.82	0.70	0.97	0.95	0.84	0.89
SD 3	0.83	− 0.21	0.49	0.95	0.42	0.28	0.89	0.51	0.96	0.95	0.73	0.87
SD 4	0.87	− 0.19	0.52	0.91	0.58	0.30	0.92	0.20	0.92	0.94	0.68	0.86
SD 5	0.89	− 0.07	0.53	0.87	0.76	0.35	0.93	− 0.09	0.86	0.92	0.75	0.86
SD 6	0.87	0.13	0.48	0.81	0.82	0.48	0.93	− 0.09	0.73	0.89	0.80	0.85
FWHM	0.97	− 0.19	− 0.52	0.88	0.41	0.07	0.55	0.41	− 0.37	0.85	0.86	0.18

IHD, ischemic heart disease; NICM, dilated/non-ischemic cardiomyopathy; HCM, hypertrophic cardiomyopathy; SD, standard deviation, FWHM, full width at half maximum.

## Discussion

In the present study we compared various LGE quantification methods using both dark- and bright-blood LGE images in different groups of patients, including those with IHD, NICM, and HCM. This study has three main findings: (1) Different LGE quantification methods, including the semi-automated SD and FWHM methods, can be applied to dark-blood LGE images similarly as to bright-blood LGE images, (2) Dark-blood LGE showed consistently more enhancement when compared with bright-blood LGE in patients with IHD, and (3) In patients with non-ischemic scar (both NICM and HCM groups), dark-blood and bright-blood LGE demonstrated a similar extent of enhancement by any of the quantification methods evaluated.

Different methods for the quantification of LGE have been proposed, validated, and compared in previous studies using conventional bright-blood LGE. A number of early experimental studies, with histopathology as reference standard, have used thresholds of 2^18–20^ or 3^21,22^ SDs for bright-blood LGE images in settings of both acute and chronic MI. Many later studies, however, showed that signal intensity thresholding with a threshold of 2 and 3 SDs may lead to a significant overestimation of infarct size. Bondarenko et al. investigated a chronic MI setting, and compared manual contouring and signal intensity thresholding (2 to 6 SDs) for quantifying enhancement on bright-blood LGE images^23^. They found that when using the 5 SD method, infarct size did not differ from manual contouring. The 6 SDs method showed a non-significant underestimation, while the 2 through 4 SDs methods all showed a significant overestimation of the extent of scar. In a study of acute MI, Vermes et al. also found a threshold of 5 SDs to agree best with manual contouring in bright-blood LGE images^24^. In a study by Flett et al., manual contouring and signal intensity thresholding (using 2 to 6 SDs and the FHWM method) were compared in bright-blood LGE images in a cohort of both chronic and acute MI patients^17^. No significant difference was observed between manual contouring and signal intensity thresholding using 5 and 6 SDs in chronic MI patients, and using 6 SDs in acute MI patients. Gruszczynska et al. demonstrated that a threshold of 4 SDs agreed best in an animal model of acute MI using histopathology as reference standard^25^. Manual contouring was found to underestimate the infarcted myocardium by − 2 to − 3%, therefore agreeing best with a threshold of 5 or 6 SDs which is in line with the findings of both Vermes et al.^24^ and Flett et al.^17^ These past results in studies evaluating scar quantification using bright-blood images LGE in a setting of MI are in line with the findings of the present study, demonstrating that a threshold of 5 and 6 SDs also agreed best with manual thresholding using bright-blood LGE images in the IHD group.

LGE quantification is assuming greater importance in the risk stratification of non-ischemic cardiomyopathies, such as HCM^26^. In NICM patients, LGE extent was found to be relevant for predicting all-cause mortality, sudden cardiac death, heart failure, and cardiovascular-related events^27^. These observations made it sensible to assess performance of quantification methods in scar of non-ischemic nature, which was explored using conventional bright-blood LGE technique in several studies in the past. Mikami et al. assessed the performance of threshold-based LGE quantification (2, 3, and 5 SDs) versus manual thresholding in evaluating mid-wall septal fibrosis and its ability to predict outcomes in DCM patients^28^. Semi-automated thresholding using a threshold of 3 SDs showed the best correlation with manual thresholding. Flett et al. evaluated manual contouring and semi-automated scar quantification in HCM patients and found that both a threshold of 6 SDs and the FWHM method did not significantly differ from manual contouring in bright-blood LGE images^17^. Other studies focusing on scar quantification in bright-blood LGE images in HCM patients found similar results. Spiewak et al. compared manual contouring, signal intensity thresholding (1 to 6 SDs and the FWHM method), and thresholding using peak remote myocardium^29^. No difference between the 6 SD threshold, FWHM and manual contouring was found, with the best agreement to manual contouring being a threshold of 6 SDs. Harrigan et al. also found a threshold of 6 SDs to agree best with manual contouring, while an overestimation was observed when using lower thresholds^30^. Also when using bright-blood LGE in the HCM group in the present study, a threshold of 6 SDs agreed best with manual thresholding.

So far, however, quantification of LGE has mainly been applied to conventional bright-blood LGE images with hardly any literature available on the performance of such methods for the increasingly used dark-blood LGE techniques, in particular in scar of non-ischemic origin. In a conference abstract, Kotecha et al. evaluated infarct size in 39 patients with IHD by comparing an T_2_-prepared black-blood LGE technique with standard bright-blood PSIR LGE imaging^31^. LGE quantification methods used in this study included manual contouring, signal intensity thresholding (5 and 6 SDs, and the FWHM method), and Otsu’s method^32^. It was found that a threshold of 6 SDs agreed best with manual contouring in bright-blood LGE images, while a threshold of 5 SDs agreed best in dark-blood LGE images. In this cohort of IHD patients, dark-blood LGE found significantly more scar than conventional bright-blood LGE based on manual contouring. The results of this paper support our study findings even though were obtained with a different dark-blood LGE sequence aimed at achieving the same goal. More recently, Nies et al. evaluated the performance of various semi-automated techniques/methods and manual contouring for quantification of MI size using both conventional bright-blood and dark-blood LGE in animal model with histopathology as reference standard^16^. Among the assessed semi-automated quantification methods of signal intensity thresholding (3 to 8 SDs and the FWHM method), they found the best agreement with histopathology using a signal intensity threshold of 5 SD for both bright-blood LGE and dark-blood LGE. When using manual contouring as reference, optimal agreement was found when using a signal intensity threshold of 6 SDs for bright-blood LGE and 5 SDs for dark-blood LGE, which is in agreement with the study of Kotecha et al. However, important to note is that manual contouring using bright-blood LGE led to a significant underestimation of scar, hence agreed better with a higher threshold, while a near perfect agreement between manual contouring and histopathology was found when using dark-blood LGE. This significant underestimation of scar using bright-blood LGE was also demonstrated in two other recent studies^6,12^ that evaluated infarct size (using manual contouring only) in dark-blood LGE images using histopathology as reference standard. In both of these studies, dark-blood LGE showed a near perfect agreement with histopathology.

Taken together, this and previous studies demonstrate that dark-blood LGE techniques not only consistently delineate more extensive scar than bright-blood LGE images in patients with IHD, but also that these measurements are more accurate in comparison with histopathology.

One should be aware that dark-blood LGE detects significantly more ischemic scar using manual contouring than bright-blood LGE, and thereby different thresholds may apply for obtaining best agreement with manual contouring. In this study, where for the first time the performance of different quantification methods was assessed using dark-blood LGE in non-ischemic pathologies, no significant differences between both LGE methods were found. For these non-ischemic pathologies (NICM and HCM group), where the scar is within the myocardium itself and does not abut the blood pool, no particular advantage or disadvantage was indeed expected for dark-blood LGE as it was designed for solving a problem that does not exist for these pathologies. Although these results suggest existing thresholds may therefore be used interchangeably, it was found however that in the NICM and HCM groups different thresholds agreed best with manual thresholding for the different LGE methods.

### Limitations

A limitation of the current study, however, is the lack of histopathology, and instead manual thresholding was used as reference standard similar to the majority of other studies on scar quantification. Inherently, the measurements may have subjectivity as illustrated by the inter-observer variability, in particular in the NICM and HCM groups. Although the external validity of the present findings is less robust given this single-center nature of this study with a relatively small number of patients for each subgroup, studies at other centers investigating the quantification of ischemic^16^ and non-ischemic^33^ scar using dark-blood LGE compared to conventional bright-blood LGE found similar results. Finally, high-resolution 3D LGE imaging is increasingly used for accurate substrate detection in the work-up for ablation therapy and implantable cardioverter-defibrillator (ICD) implantation. Although the used dark-blood LGE method in this study can also be used for high-resolution 3D LGE imaging^34^, only 2D LGE was used to allow for comparison with standard bright-blood LGE given the longer scan times for such 3D LGE imaging.

## Conclusions

In conclusion, semi-automated LGE quantification using dark-blood LGE images is feasible in both patients with ischemic and non-ischemic scar patterns. Given the proven advantage of dark-blood LGE imaging in patients with ischemic heart disease and no disadvantages in patients with non-ischemic scar, dark-blood LGE may be adopted into clinical practice without compromising on quantification. Since the dark-blood LGE method used in this study (blood-nulled PSIR LGE) is readily and widely available on any scanner, a convenient and readily implementation in routine clinical practice is guaranteed.

## Data Availability

The data that support the findings of this study are available from the corresponding author upon reasonable request.

## Abbreviations

bSSFP: Balanced steady-state free-precession
CMR: Cardiac magnetic resonance
DCM: Dilated cardiomyopathy
FWHM: Full width at half maximum
HCM: Hypertrophic cardiomyopathy
ICC: Intraclass correlation coefficient
ICD: Implantable cardioverter-defibrillator
IHD: Ischemic heart disease
IR: Inversion-recovery
LGE: Late gadolinium enhancement
LV: Left ventricular/left ventricle
MI: Myocardial infarction
NICM: Non-ischemic cardiomyopathy
PSIR: Phase-sensitive inversion-recovery
RV: Right ventricular/right ventricle
SCMR: Society for cardiovascular magnetic resonance
SD: Standard deviation
TE: Echo time
TR: Repetition time
TI: Inversion time
